# Population pharmacokinetics, exposure-safety, and immunogenicity of atezolizumab in pediatric and young adult patients with cancer

**DOI:** 10.1186/s40425-019-0791-x

**Published:** 2019-11-21

**Authors:** Colby S. Shemesh, Pascal Chanu, Kris Jamsen, Russ Wada, Gianluca Rossato, Francis Donaldson, Amit Garg, Helen Winter, Jane Ruppel, Xin Wang, Rene Bruno, Jin Jin, Sandhya Girish

**Affiliations:** 1 Department of Clinical Pharmacology Oncology, Genentech Inc., South San Francisco, CA 94080 USA; 2 Clinical Pharmacology, Modeling and Simulation, Genentech/Roche, Marseille, France; 3 Certara Strategic Consulting, Princeton, NJ USA; 4 Clinical Science, F. Hoffmann-La Roche Ltd, Basel, Switzerland; 5 Safety Science, Roche Products Ltd, Welwyn Garden City, UK; 6 Present address: Quantitative Pharmacology and Disposition, Seattle Genetics, South San Francisco, CA USA; 7 Bioanalytical Sciences, Genentech Inc., South San Francisco, CA USA

**Keywords:** Atezolizumab, Cancer immunotherapy, Clinical pharmacology, Exposure-safety, Immune checkpoint inhibitor, Pediatric oncology, Population pharmacokinetics

## Abstract

**Background:**

The iMATRIX-atezolizumab study was a phase I/II, multicenter, open-label study designed to assess the safety and pharmacokinetics of atezolizumab in pediatric and young adult patients. We describe the pharmacokinetics (PK), exposure-safety, and immunogenicity of atezolizumab in pediatric and young adults with metastatic solid tumors or hematologic malignancies enrolled in this study.

**Methods:**

Patients aged < 18 years (*n* = 69) received a weight-adjusted dose of atezolizumab (15 mg/kg every 3 weeks [q3w]; maximum 1200 mg); those aged ≥ 18 years (*n* = 18) received a flat dose (1200 mg q3w). A prior two-compartment intravenous infusion input adult population-PK (popPK) model of atezolizumab was used as a basis to model pediatric data.

**Results:**

A total of 431 atezolizumab serum concentrations from 87 relapse-refractory pediatric and young adult patients enrolled in the iMATRIX-atezolizumab study were used for the popPK analysis. The dataset comprised predominantly patients aged < 18 years, including two infants aged < 2 years, with a wide body weight and age range. The clearance and volume of distribution estimates of atezolizumab were 0.217 L/day and 3.01 L, respectively. Atezolizumab geometric mean trough exposures were ~ 20% lower in pediatric patients versus young adults; this was not clinically meaningful as both groups achieved the target concentration (6 μg/mL). Safety was similar between pediatric and young adult patients with no exposure-safety relationship observed. Limited responses (4/87) precluded an exposure-response assessment on outcomes. A comparable rate (13% vs 11%) of atezolizumab anti-drug antibodies was seen in pediatric and young adult patients.

**Conclusions:**

These findings demonstrate a similar exposure-safety profile of atezolizumab in pediatric and young adult patients, supportive of weight-based dosing in pediatric patients.

**Trial registration:**

NCT02541604.

## Background

Pediatric patients with advanced cancers are sometimes faced with resistant or recurrent disease that cannot be cured by surgery, chemotherapy, or radiation. To improve outcomes, alternate treatment approaches such as immunotherapies, targeted therapies, and combination treatment paradigms have been investigated [1]. Immune checkpoint inhibitors (ICI) are a widely-researched class of anticancer agents, with at least 2250 trials in adults and 11 trials in children either ongoing or completed [2, 3]. While the use of these therapies has been transformative in adults, pediatric research of tolerable and efficacious ICI is limited. Gaps in knowledge on dosing, safety, and efficacy has led to substantial challenges for drug development, which has given rise to off-label use of some drugs in children [4–8].

Pediatric studies have become a keen focus of health authorities, including the US Food and Drug Administration and European Medicines Agency, which mandate pediatric studies and investigational plans to explore the use of new medicines to cover all relevant pediatric age groups, in the absence of a waiver [9, 10]. When a similar exposure-response relationship is expected, pediatric bridging studies aim to determine dosing regimens leading to similar target exposures as those observed in adults, with pediatric pharmacokinetic (PK), safety, and efficacy data collected across the appropriate age and developmental spectrum [11, 12]. An increased understanding of pediatric pharmacometrics is integral to drug development, and clinical data across multiple tumor types, body weights, age groups, including a well characterized population-PK (popPK) analysis, and relevant exposure-response analysis can assist with optimizing pediatric dosing of modern ICI drugs. [13–16].

Atezolizumab is a monoclonal antibody (mAb), under investigation as an ICI therapy, which targets programmed cell death-ligand 1 (PD-L1) to block interaction with its receptors, programmed cell death-1 (PD-1) and B7.1. Atezolizumab is approved for use in several adult tumor types in the USA, EU, and other countries [17–20]. Knowledge on the quantitative clinical pharmacology characteristics of atezolizumab in adults is extensive, yet data in pediatric patients are lacking. Response of pediatric patients with solid tumors to ICI is under investigation, and available data indicate different response patterns to those seen in certain adult tumors [21–23]. Furthermore, factors such as total body water, volume of distribution (V), cardiac output, tissue perfusion rates, and ontogeny of neonatal Fc receptor (FcRn) can impact the exposure and pharmacology of mAbs in younger children, which could be important in assessing ICIs in pediatric patients [24–26].

Given the wide range of body weights and differential growth rates expected in pediatric patients, a weight-adjusted dose of atezolizumab (15 mg/kg every 3 weeks [q3w]) was considered appropriate for clinical investigation. We aimed to achieve exposures close to and within the clinical range of those in adults, which have been established in approved indications and have shown no exposure-response relationships of safety and efficacy [27, 28]. Furthermore, a target minimum exposure of atezolizumab was set at 6 μg/mL based on tissue distribution data in tumor-bearing mice, target-receptor occupancy in the tumor, and observed atezolizumab PK in humans. Assumptions made in establishing the target exposure level for atezolizumab included a 95% tumor-receptor saturation needed for efficacy [29].

Here, we summarize key clinical pharmacology data from the phase I/II iMATRIX-atezolizumab study (NCT02541604, study GO29664), which evaluated the safety, tolerability, PK, immunogenicity, and preliminary efficacy of atezolizumab monotherapy in pediatric and young adult patients with solid tumors [30].

## Methods

### Study design

The iMATRIX-atezolizumab study (NCT02541604) was a phase I/II, multicenter, open-label study to assess the safety and PK of atezolizumab in pediatric patients and young adults. The study enrolled patients with solid tumors with known or expected PD-L1 pathway involvement for which prior treatment was proven to be ineffective or intolerable, and for whom there was no curative standard-of-care treatment. Patients with Hodgkin lymphoma (HL), non-Hodgkin lymphoma (NHL), or other rare tumors with/without documented expression of PD-L1 on tumor cells or immune infiltrating cells were eligible. Patients with history of any autoimmune disease were excluded. However, patients with a history of autoimmune-related hypothyroidism on a stable dose of thyroid-replacement hormone, or those with controlled Type 1 diabetes mellitus on a stable insulin regimen were eligible. Patients received atezolizumab q3w using a weight-adjusted dose at 15 mg/kg for patients aged < 18 years (maximum dose 1200 mg), and a flat dose of 1200 mg for patients aged ≥ 18 years. Atezolizumab was administered by intravenous infusion on day 1 of each cycle, with an infusion duration of 60 min in cycle 1 and 30 min in subsequent cycles. The study was conducted in accordance with the Declaration of Helsinki and Good Clinical Practice Guidelines, following approval by the ethics board in each institution. Informed consent was obtained from each patient or each patient’s authorized representative.

### Pharmacokinetic and immunogenicity sampling and analytical methods

The PK/anti-drug antibody (ADA) sampling schedule following atezolizumab administration was designed to describe its distribution, elimination, and immune response. The sparse PK/ADA sampling schedule was used to assess PK and ADA after single and repeated dosing. Pharmacokinetic and ADA sampling of atezolizumab was performed at the end of infusion on day 1 of cycles 1 and 4 (PK only), and C_min_ and ADA were collected prior to infusion on day 1 of cycles 2, 3, 4, 8, 12, 16, and every 8 cycles thereafter. Atezolizumab was quantified by enzyme-linked immunosorbent assay (ELISA). The lower limit of quantification (LOQ) for the atezolizumab assay in human serum was 60 ng/mL. Samples for ADA analysis were evaluated using a bridging ELISA assay with positive samples in screening further confirmed by titer. Further details for PK and immunogenicity assays have been reported previously [27].

### Data source

Exploration and visualization of the data, as well as descriptive statistics, were performed using R v3.3.1 with additional CRAN packages. The dataset included 520 samples; 81 samples that occurred prior to the first dose were below the LOQ and were excluded. Data manipulation was limited to flagging data records not used for analysis, imputing missing variables to median values, and excluding patients with no dose information (*n* = 1). Missing covariate values were imputed to median values for continuous covariates or to the most frequent category for categorical covariates.

### PopPK model

The popPK analysis was performed using a non-linear mixed-effects modeling approach with NONMEM v7.3 (ICON Development Solutions, Ellicott City, Maryland, USA) in conjunction with Perl-Speak-NONMEM (PsN) (v3.7.6, Uppsala University, Uppsala, Sweden). A prior two-compartment intravenous infusion input adult popPK model of atezolizumab was used as a basis to model pediatric data. The typical clearance (CL; L/day) of atezolizumab for an adult patient *i* was:

$$ {\mathrm{CL}}_i=\left(0.200\bullet {\left(\frac{ALBU_i}{40}\right)}^{-1.12}\bullet {\left(\frac{BWT_i}{77}\right)}^{0.808}\bullet {\left(\frac{TUM_i}{63}\right)}^{0.125}\right)\bullet \left(1.159\  if\  ADA\  is\ positive\right) $$

*BWT: Body weight (kg); ALBU: Albumin (g/L); TUM: Tumor burden (mm); ADA: Post-baseline status of anti-drug antibodies.*

The typical volume of the central compartment (V1; L) and volume of the peripheral compartment (V2; L) of atezolizumab for an adult patient *i* were:

$$ V{1}_i=\left(3.28\bullet {\left(\frac{BWT_i}{77}\right)}^{0.559}\bullet {\left(\frac{ALBU_i}{40}\right)}^{-0.350}\right)\bullet \left(0.871\  if\ female\right) $$

$$ V{2}_i=3.63\bullet \left(0.728\  if\ female\right) $$

An extensive list of covariates reflecting cancer status/type, organ dysfunction, and race/region tested in the prior adult PK model were not re-tested in the pediatric and young adult model. To provide consistency between the adult and pediatric popPK analyses, the adult popPK model was fitted to the pediatric PK data, utilizing the same structure, but re-estimating each parameter. Proportional changes (i.e., for ADA and sex) in the pediatric and young adult model were parameterized *θ*.*cov*, where *θ* indicates the proportional change and *cov* was either ADA or sex (both coded 0 or 1); this differed from the prior adult model.

### Model diagnostics

The performance of the model was evaluated using standard diagnostic plots to evaluate the observed dependent variable (atezolizumab concentration) versus population predictions, dependent variable versus individual predictions, conditional weighted residuals (CWRES) versus population predictions, CWRES versus time, quantile-quantile plot of CWRES, random effect distributions, and correlations of random effects between parameters. The predictive performance of the popPK model was also evaluated with a prediction-corrected visual predictive check with 500 replicates [31, 32].

### Derivation of exposure metrics

Individual empirical Bayesian estimates of PK parameters were used to compute atezolizumab exposure variables based on the nominal dose regimen including area under the curve (AUC), maximum concentration (C_max_), and minimum concentration C_min_, in cycle 1 and at steady-state. The cycle 1 and steady-state PK profile for each individual based on the starting dose was simulated using individual empirical Bayesian estimates of PK parameters based on the final model. The following time points were used for simulations: 0, every 0.01 day for the first 3 days, every 0.5 days until 21 days post dose, and 20.99 days post dose at cycle 1, and a similar schedule at steady-state (cycle 10). Atezolizumab exposure metrics including C_max_, C_min_, and AUC (cycle 1) were derived from the simulated individual PK profiles, and AUC at steady-state was derived as dose/CL. The resulting metrics were compared and stratified by age group using box-plots.

### Exposure-safety analysis

The exposure-response analysis of safety was conducted using data from all atezolizumab-treated patients for whom exposure data were available. p(AE) is the observed probability of an adverse event (AE) versus atezolizumab AUC in cycle 1. Exposure levels of atezolizumab were binned based on the quantiles of the log-transformed AUC. A mean curve obtained from averaging each exposure record in the data set and binning boundaries by quartiles of exposure was established. Bootstrapped replicates (*n* = 100) were used to plot the 90% confidence band for the mean fit curve. The overall analysis represented findings across 69 pediatric patients.

### Efficacy

The primary efficacy outcome measures were objective response rate (ORR) and progression-free survival (PFS). ORR was defined as the proportion of patients with measurable disease at baseline who achieved a complete or partial response, with response on two consecutive occasions ≥ 4 weeks apart, as determined by the investigator using Response Evaluation Criteria in Solid Tumors (RECIST) v1.1. PFS was defined as the time from initiation of study drug to the first documented occurrence of disease progression, as determined by the investigator using RECIST v1.1 criteria.

### Immunogenicity

The presence of ADAs to atezolizumab during the study, relative to baseline, and in relationship to the serum concentration of atezolizumab at specified time points was determined. Characterization of immunogenicity was performed for all patients with at least one ADA assessment. Patients were considered ADA positive if they were ADA negative or had missing baseline data but developed an ADA response following study drug exposure, or if they were ADA positive at baseline and the titer of one or more post-baseline samples was ≥ 0.60 titer-units greater than that of the baseline sample. Patients were considered ADA negative if they were ADA negative or had missing baseline data and all post-baseline samples were negative, or if they were ADA positive at baseline but did not have any post-baseline samples with a titer that was ≥ 0.60 titer-units greater than that of the baseline sample.

## Results

### Patient demographics

A total of 431 atezolizumab serum concentrations from 87 relapsed-refractory pediatric and young adult patients enrolled in the iMATRIX-atezolizumab study were used for the popPK analysis. The dataset comprised predominantly patients aged < 18 years, including two infants aged < 2 years, with a wide body weight (8.7–154 kg) and age range (7 months–29 years). Median age and weight was 12 years and 38.9 kg, respectively, across the 69 pediatric patients, and 22 years and 61.0 kg, respectively, across the 18 young adults (Table 1 and Additional file 1: Figure S1).

**Table 1:** Patient baseline demographic and clinical characteristics

Covariate	Infants < 2 years *n* = 2		Children 2 to < 12 years *n* = 29		Adolescents 12 to < 18 years *n* = 38		Young adults ≥ 18 years *n* = 18	
	*n* (%)	Median (min–max)	*n* (%)	Median (min–max)	*n* (%)	Median (min–max)	*n* (%)	Median (min–max)
Age, years	2	1 (0.6–1.5)	29	7 (2–11)	38	15 (12–17)	18	22 (18–29)
Body weight, kg	2	9.1 (8.7–9.5)	29	22.5 (12.0–74.4)	38	51.1 (28.2–105)	18	61.0 (46.2–154)
Albumin, g/L	2	33 (30–35)	28^a^	41 (23–46)	38	41 (29–49)	18	39 (27–47)
Tumor burden, mm	2	59 (35–83)	23^b^	55 (15–301)	28^d^	78 (11–208)	15^e^	120 (10–258)
Female	1 (50)	–	14 (48)	–	16 (42)	–	9 (50)	–
ADA positive	0 (0)^a^	–	5 (17)^c^	–	4 (11)^e^	–	2 (11)^a^	–
No. of tumor types present	2	1	29	12	38	15	18	10
Lansky/Karnofsky PS	2	90 (90–90)	29	100 (60–100)	38	90 (70–100)	18	95 (70–100)

*Abbreviations*: *ADA* Anti-drug antibodies, *PS* Performance status

^a^*n* = 1, ^b^*n* = 6, ^c^*n* = 5, ^d^*n* = 10, ^e^*n* = 3 missing covariates were imputed to the median value

Descriptive statistics of patient characteristics and covariates by age group are summarized in Table 1. Baseline demographics were balanced in terms of gender. Although the sample size was limited, no apparent difference in albumin or ADA response to atezolizumab by age was seen (*P* > 0.05). Multiple tumor types were present including Ewing sarcoma, neuroblastoma, non-rhabdomyosarcoma soft tissue sarcoma, osteosarcoma, rhabdomyosarcoma, Wilms’ tumor, HL, NHL, malignant rhabdoid tumor, atypical teratoid/rhabdoid tumor, and other rare tumors. The number of tumor types was diverse across the age groups, with median tumor burden increasing with age. The majority of patients had a Lansky/Karnofsky performance score ≥ 80%.

### Pediatric and young adult popPK model

A pediatric and young adult model was established with the current study data utilizing the same structure as the adult popPK model to allow for consistency, while re-estimating each parameter. The prior adult model was a two-compartment model with an intravenous infusion input.

Parameter estimates from the modeling are displayed in Table 2. Parameters were estimated with good precision. Parameter estimates for CL and V of 0.217 L/day, and 3.01 L, respectively, including covariate effects, were generally in line with the prior adult popPK model. Two exceptions were the estimates for V2 and inter-compartmental clearance (Q), which were not weight-normalized, and decreased in pediatric patients. As a sensitivity analysis, the inclusion of weight and age on V2 and Q resulted in estimates closer to, but still less than, those achieved in adults. Sex effects had minimal impact on the objective function. Between-subject and residual variability were acceptable given the relatively small numbers of patients and sparse PK sampling.

**Table 2:** Parameter estimates in pediatric and young adult patients

Parameter	Pediatric/young adult model			Adult model		
	Estimate	%RSE	Shrinkage (%)	Estimate	%RSE	Shrinkage (%)
CL (L/day)	0.217	5		0.200	2	
V1 (L)	3.01	4		3.28	2	
V2 (L)	1.36	11		3.63	4	
Q (L/day)	0.183	18		0.546	8	
Weight on CL	0.795	8		0.808	8	
Albumin on CL	−1.18	20		−1.12	10	
Tumor burden on CL	0.122	35		0.125	17	
Positive ADA on CL	1.23	8		1.16	25	
Weight on V1	0.766	8		0.559	8	
Albumin on V1	−0.566	29		−0.350	21	
Sex (female) on V1	NE	–		− 0.129	16	
Sex (female) on V2	NE	–		−0.272	16	
Proportional residual variance	0.051	32	12	0.043	7	9
Additive residual variance	68.9	109	12	16.6	39	9
BSV for CL	0.0458	33	21	0.0867	9	9
BSV for V1	0.0140	65	43	0.0328	18	17
BSV for V2	0.311	63	39	0.114	25	33
Correlation of BSVs for CL, V1	0.510	–		0.341		
Correlation of BSVs for CL, V2	NE	–		−0.236		
Correlation of BSVs for V1, V2	NE	–		0.434		
Objective function	3637			40,748		

*Abbreviations*: *%RSE* Percent relative standard error, *ADA* Anti-drug antibody, *BSV* Between-subject variability, *CL* Clearance, *NE* Not evaluated, *Q* Inter-compartmental clearance, *V1* Volume of the central compartment, *V2* Volume of the peripheral compartment

**Fig. 1:**
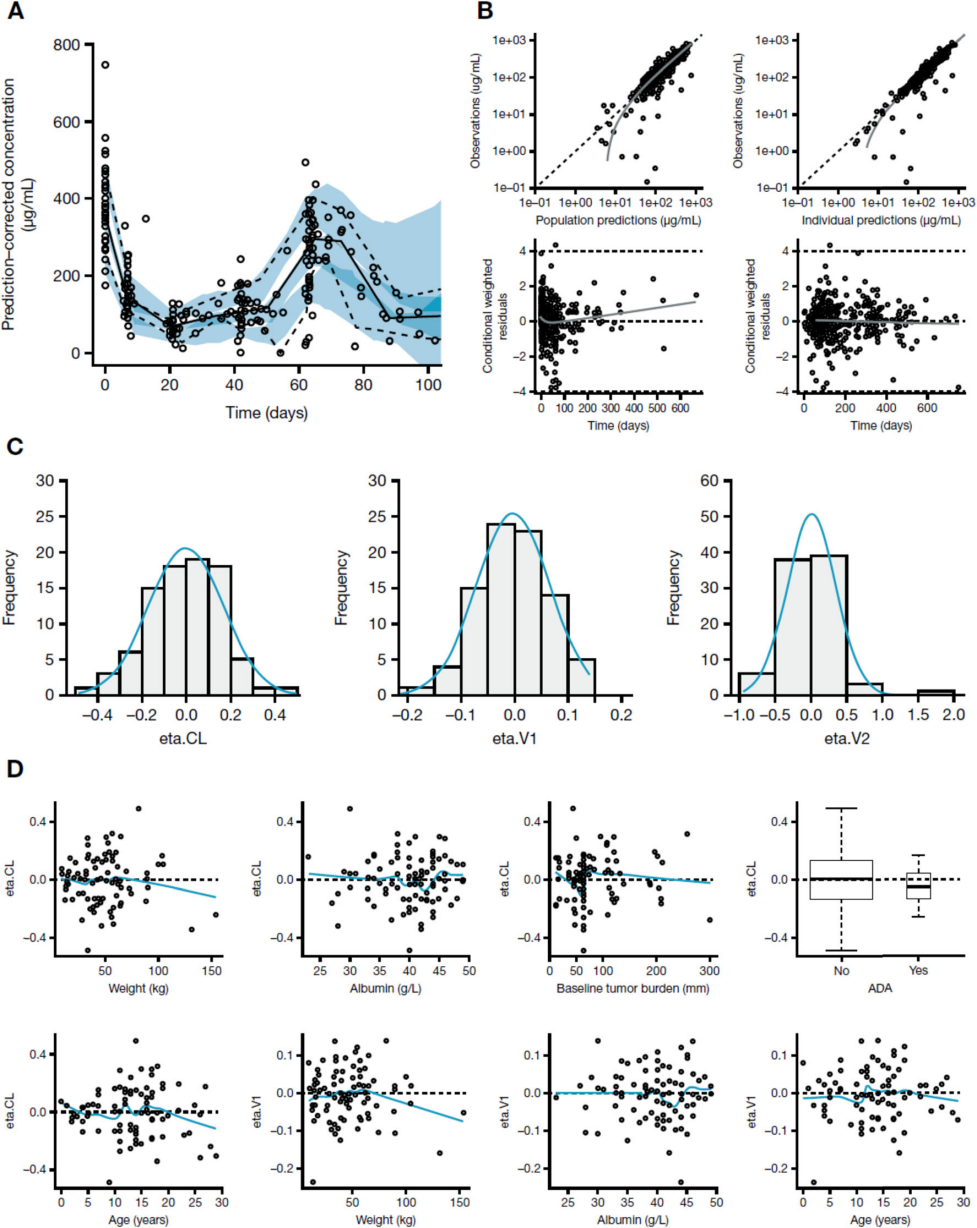
(**a**) Prediction-corrected visual predictive check, (**b**) goodness of fit diagnostic plots, (**c**) Eta distributions, and (**d**) random effect correlations to covariates. Prediction-corrected visual predictive check (**a**): the gray solid and dashed lines represent the observed median and the 10th and 90th percentiles, respectively, while the two shades of blue represent overlap between the empirical 95% prediction intervals. Goodness of fit diagnostic plots (**b**): the gray solid line indicates fitted values from a nonparametric smoother. Dashed lines indicate the line of unity (top plots), or zero lines and boundary lines for conditional weighted residuals (bottom). Eta distributions (**c**): the blue solid line represents a density curve. Random effect correlations to covariates (**d**): for continuous covariates, the blue solid line represents fitted values from a nonparametric smoother. The dashed line indicates the zero line, the box-plot indicates the median and interquartile range (25th to 75th percentile), the whiskers indicate 1.5 times the interquartile range. Abbreviations: *ADA* anti-drug antibody, *CL* clearance, *V1* volume of the central compartment, *V2* volume of the peripheral compartment

Graphical evaluations of the final popPK model are displayed in Fig. 1. The plots suggest that the model is adequate with respect to structure and covariate parameterizations. In particular, relationships of the random effects for CL and V (eta.CL and eta.V1) did not show any bias with age (smooth curve showing a horizontal linear relationship around zero) (Fig. 1d) suggesting that the body weight effects in these parameters captured the difference between adults and pediatric patients. The prediction-corrected visual predictive check (Fig. 1a) suggested that the model captured the central tendency and the variability in PK. Given the interest in body surface area (BSA)-based dosing for pediatric patients, a plot of the random effects of CL and V1 by BSA was also explored (Additional file 2: Figure S2). No bias was revealed, suggesting that covariates including body weight in the model also account for changes in BSA, highlighting the appropriateness of weight-based dosing.

### Exposure metrics

**Fig. 2:**
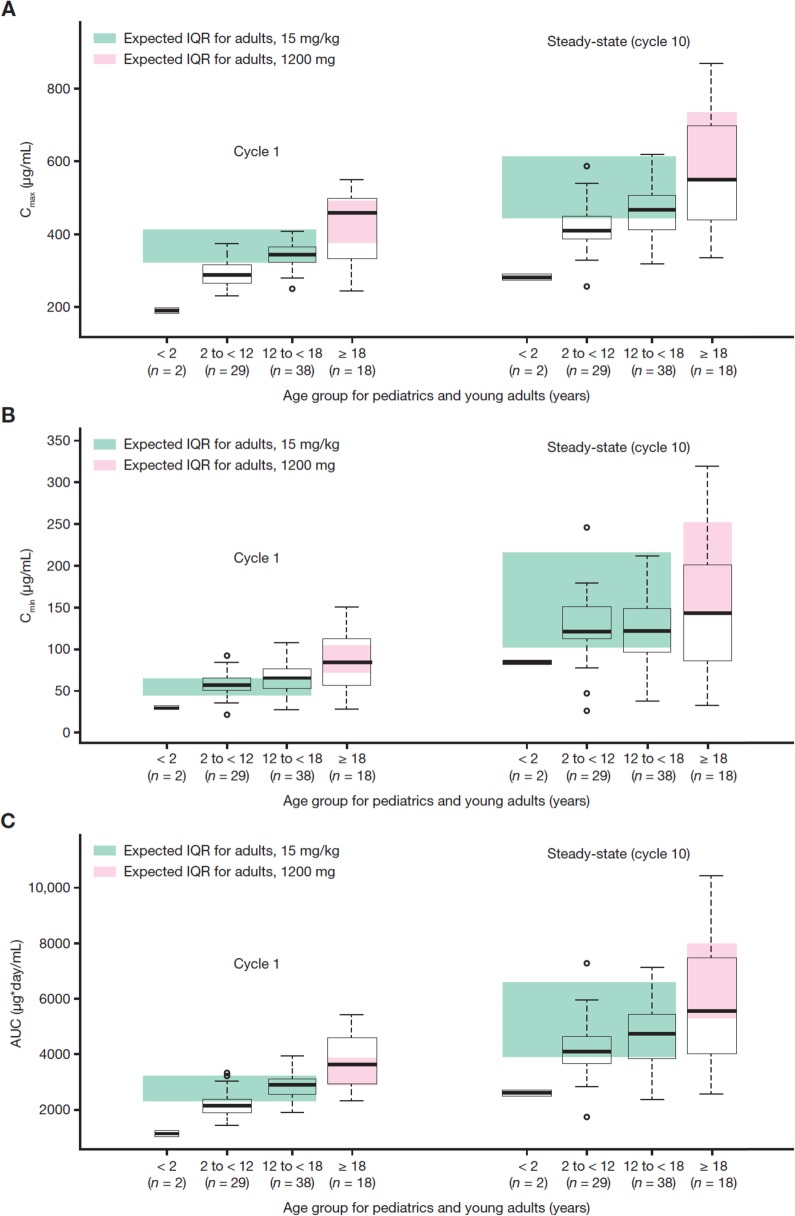
Cycle 1 and steady-state (cycle 10) exposure metrics by age group: (**a**) C_max_, (**b**) C_min_, and (**c**) AUC. Expected interquartile range (IQR) from simulated distributions (*n* = 1000) based on reported geometric means and %CVs. The box-plots indicate the median and IQR (25th to 75th percentile). The whiskers indicate 1.5 times the IQR. Abbreviations: *AUC* area under the curve, *C*_*mi*n_ minimum concentration, *C*_*max*_ maximum concentration

Summaries of the individual exposure metrics are displayed in Fig. 2, based on individual model predictions across the 87 patients at cycle 1 and steady-state. Overall, AUC and C_max_ increased from children to adolescents to young adults, whereas C_min_ was comparable across age groups, especially at steady-state. The expected inter-quartile range (IQR) of exposure in 1000 simulated adult patients (median age: 62 years) using the adult popPK model are also shown. Following the 15 mg/kg simulated regimen in adults, a median cycle 1 C_min_ of 53.0 μg/mL with an interquartile interval (Q1 and Q3) of 44.6 and 64.7 μg/mL was predicted.

**Fig. 3:**
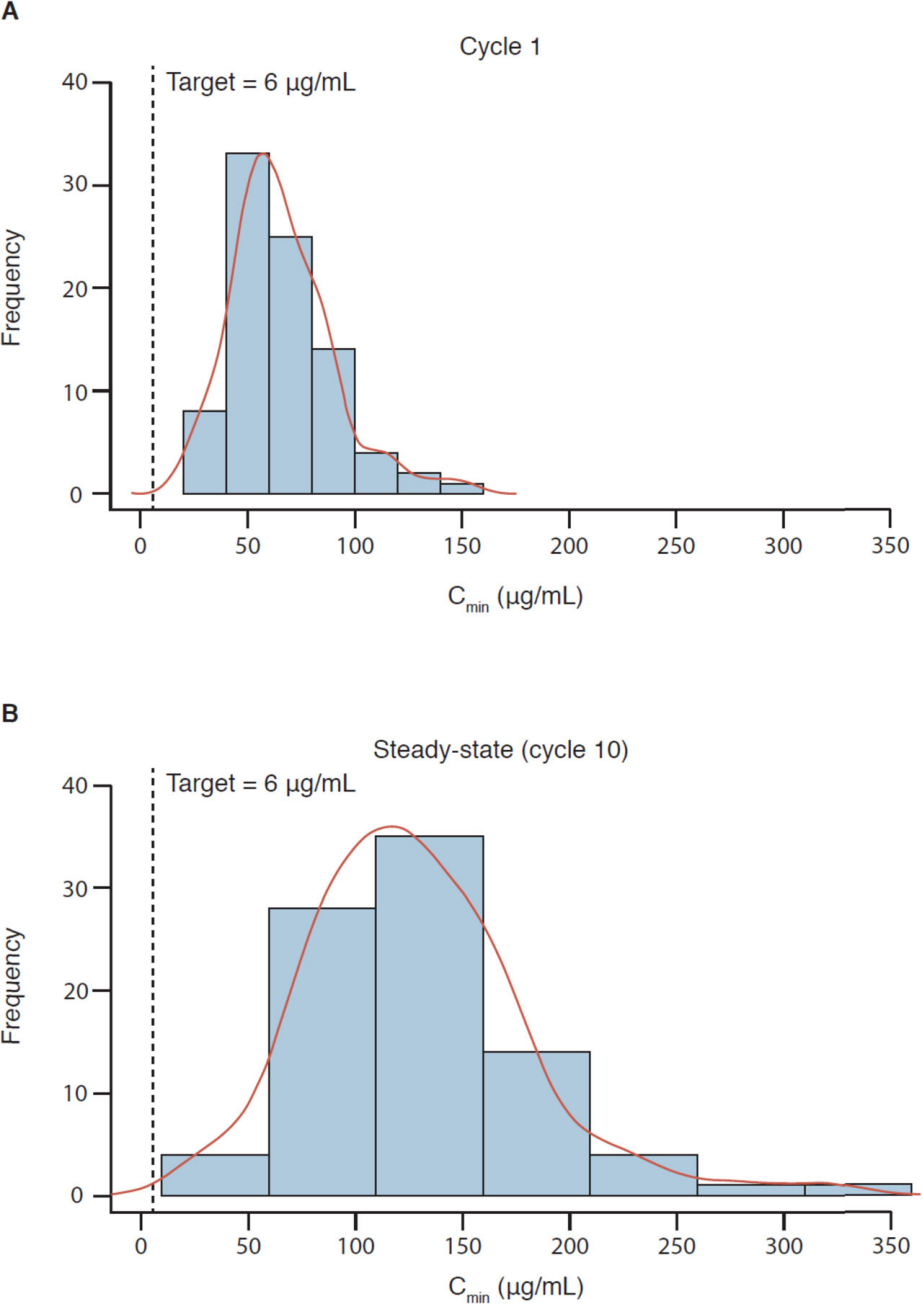
Post-hoc exposures at cycle 1 (**a**) and steady-state (cycle 10) (**b**). Exposures across 69 patients aged < 18 years (including two infants < 2 years, 29 children 2 to < 12 years, and 38 adolescents 12 to < 18 years) and 18 young adults aged 18 to < 29 years. The dotted line indicates the therapeutic target exposure of 6 μg/mL. The height of the bar represents the number of patients within that concentration range. A cumulative distribution trend (red line) is superimposed over the frequency distribution histogram. Abbreviation: *C*_*min*_ minimum concentration

The influence of body weight distribution on exposure, including summaries of C_max_, C_min_, and AUC at cycle 1 and steady-state by weight categories or tertiles, is presented in Table 3. Exposures in pediatric patients and young adults were generally consistent with exposures in adults (i.e., medians of pediatric patients and young adults were within the range of adults). Children aged 2 to < 12 years had approximately 20% lower AUC and C_max_ than adults who received 15 mg/kg atezolizumab q3w. The geometric mean (%CV) cycle 1 C_min_ of 55.9 μg/mL in 29 pediatric patients aged 2 to < 12 years, and 62.4 μg/mL in 38 adolescent patients aged 12 to < 18 years, are both generally similar (within 10–30% difference) with those seen in adults receiving a 1200 mg dose. For additional reference purposes of pediatric exposure to adults, a median (5th–95th percentile) cycle 1 C_min_ of 77.3 (40.1–132) μg/mL was simulated in 500 adult patients with various tumor types who received single-agent 1200 mg atezolizumab. Lastly, the terminal half-life of atezolizumab (~ 2–3 weeks) in pediatric patients and young adults were consistent with those estimated in adults. Variability in exposure decreased in the 2 to < 12 years group and the 12 to < 18 years group relative to the ≥ 18 years group. Results in infants have limited interpretation due to the small sample size. Figure 3 illustrates the distribution of cycle 1 and steady-state (cycle 10) C_min_ in patients aged < 18 years who received 15 mg/kg atezolizumab q3w, and in patients aged ≥ 18 years who received 1200 mg atezolizumab q3w.

**Table 3:** Predicted summary statistics (median [min–max]) of atezolizumab exposure metrics

Metric	Observation	Body weight dose (< 18 years)			Flat dose (≥ 18 years)		
		< 30 kg *n* = 23	30 to < 45 kg *n* = 21	≥ 45 kg *n* = 25	< 57 kg *n* = 6	57 to < 65 kg *n* = 6	≥ 65 kg *n* = 6
C_max_, μg/mL	Cycle 1	270 [182–349]	330 [232–375]	349 [281–407]	492 [303–541]	486 [419–549]	326 [243–390]
	Steady-state	400 [277–517]	463 [257–585]	460 [319–618]	664 [377–764]	651 [505–868]	404 [334–540]
C_min_, μg/mL	Cycle 1	55.6 [28.1–82.0]	65.0 [20.7–91.9]	65.5 [27.4–108]	97.3 [47.4–122]	98.8 [56.9–151]	57.6 [28.4–87.5]
	Steady-state	120 [77.0–181]	125 [25.4–246]	112 [37.9–211]	171 [74.1–225]	164 [86.2–319]	88.1 [32.4–149]
AUC, μg*day/mL	Cycle 1	2085 [1089–3053]	2757 [1471–3312]	2988 [1975–3954]	4268 [2396–4845]	4330 [3175–5448]	2733 [2306–3701]
	Steady-state	4045 [2536–5695]	4781 [1730–7295]	4510 [2417–7126]	6692 [3365–8125]	6574 [4276–10,405]	3861 [2593–5774]
CL, mL/day/kg	–	3.72 [2.62–5.89]	3.20 [2.06–8.67]	3.15 [2.90–6.10]	3.62 [2.61–5.20]	2.90 [1.99–4.92]	3.25 [1.70–5.16]

*Abbreviations*: *AUC* Area under the curve, *C*_*max*_ Maximum concentration, *C*_*min*_ Minimum concentration, *CL* Clearance

CL and V of atezolizumab in pediatric patients dosed by body weight, or young adults receiving a flat dose, demonstrated a consistent relationship across the wide body weight range (Additional file 3: Figure S3). Grouping pediatric patients and young adults by tertiles of body weight revealed similar elimination estimates (Table 3).

### Exposure-safety analysis

**Fig. 4:**
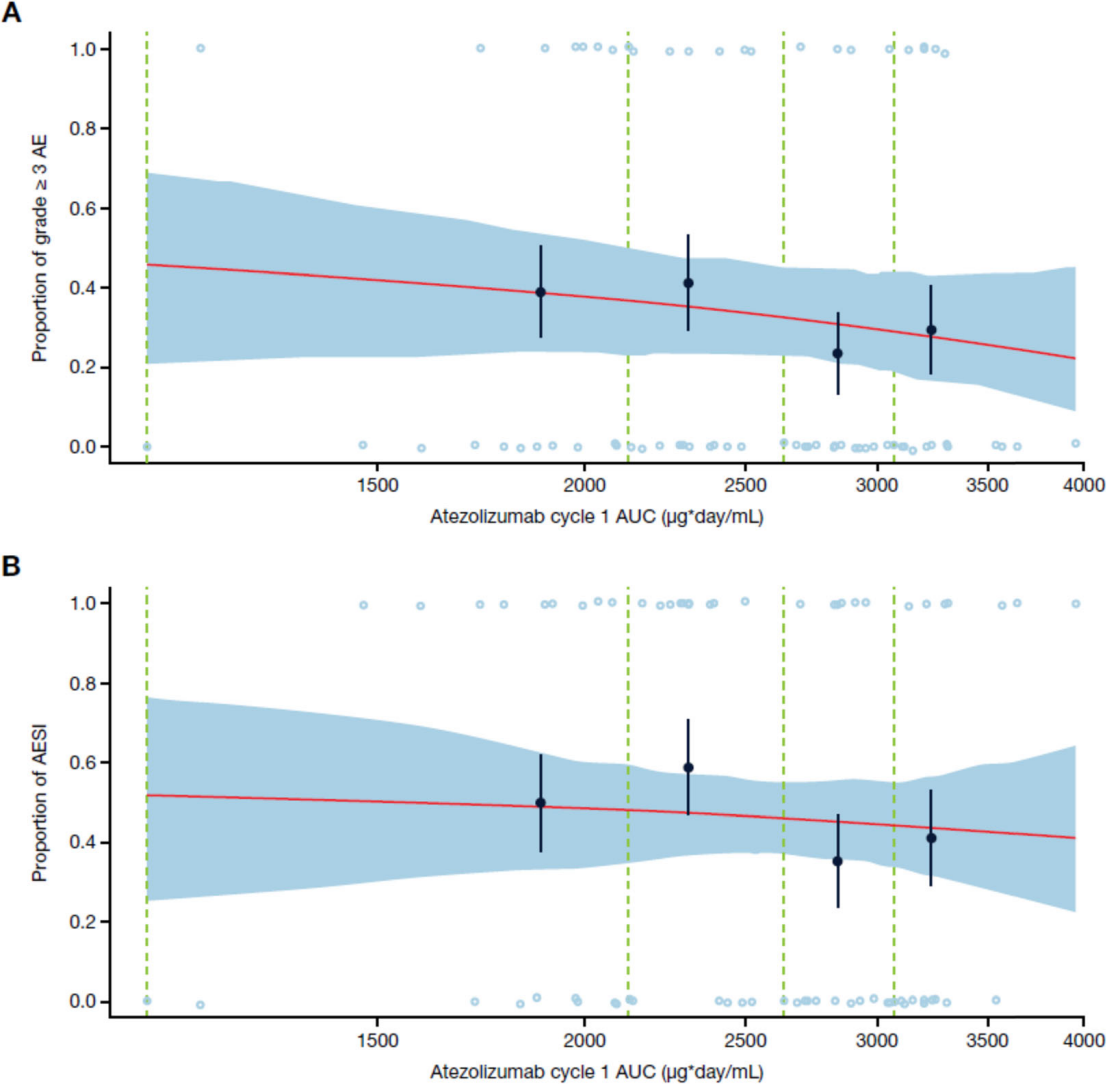
Incidence of grade ≥ 3 AEs (**a**) and any-grade AESI (**b**). AEs and AESI are displayed by open blue circles. Solid black circles with standard error bars (y-value: binned probability of having an event from observations; x-value: median exposure value within the bin). Red line: mean model fitted curve (obtained from averaging the fitted curve for each exposure record in the data set). Dashed green lines: binning boundaries. Exposure levels are binned based on the quantiles of the log transformed exposure variable levels. Blue shaded area: based on 100 bootstrap replicates, depicting the 90% confidence band for the mean model fitted curve. Plot is based on 69 patients. Abbreviations: *AE* adverse event, *AESI* adverse event of special interest, *AUC* area under the curve

The exposure-safety analysis was performed in all pediatric patients aged < 18 years with exposure data (*n* = 69). The incidence of grade ≥ 3 AEs and AEs of special interest (AESI) versus atezolizumab cycle 1 AUC is shown in Fig. 4. AESI categories included: rash, hepatitis, aspartate transferase/alanine aminotransaminase elevations, infusion-related reactions, hypothyroidism, blood thyroid-stimulating hormone increase, pancreatitis, diabetes mellitus, colitis, hyperthyroidism, and meningoencephalitis. Grade ≥ 3 AEs and all-grade AESI occurred at an incidence of 33% (events in 69 patients) and 46% (events in 69 patients), respectively. Exposure metrics within the first treatment cycle were used rather than steady-state to isolate potentially confounding factors on exposure such as time-varying clearance [33]. No exposure-response relationship with atezolizumab AUC in cycle 1 was detected.

### Efficacy

Among 87 patients, there were 4 responders (4.6%), all of whom had a partial response; 1 of these patients had malignant rhabdoid tumor, 2 had HL, and 1 had NHL. In total, 63 patients (72.4%) had disease progression, 10 (11.5%) had stable disease, 2 (2.3%) were not evaluable, and 8 (9.2%) had missing post-baseline assessments. Median PFS was 1.3 months (95% confidence interval [CI], 1.2–1.4). Overall, 63 patients were evaluable for PD-L1 expression, of whom 18% had high PD-L1 expression (IC2/3), including all of the 4 responding patients. Interpretation of atezolizumab exposure and biomarker expression with outcomes was not performed due to the low number of responders.

### Immunogenicity

Ten patients had missing ADA records, which were imputed as the median (ADA negative) for the popPK analysis. The number of imputed records by age group was: 1/2 (< 2 years), 5/29 (2 to < 12 years), 3/38 (12 to < 18 years), and 1/18 (≥ 18 years). Imputed records were not expected to impact the outcome given that < 20% of total ADA records in any given age group (apart from infants which were not interpretable) were imputed. Observed exposure and safety by ADA were interpreted using non-imputed records.

Overall, 11/87 (13%) patients were treatment-emergent ADA-positive to atezolizumab, which included 0/2 (0%) patients aged 0 to < 2 years, 5/29 (17%) patients aged 2 to < 12 years, 4/38 (11%) patients aged 12 to < 18 years, and 2/18 (11%) patients aged ≥ 18 years. The observed geometric mean peak and trough exposure of atezolizumab by ADA status in PK-evaluable patients across multiple cycles is provided in Additional file 4: Table S1. The geometric mean cycle 1 C_min_ of atezolizumab was comparable between ADA-positive (57.0 μg/mL) and ADA-negative (62.5 μg/mL) patients.

The incidence of serious AEs was broadly similar between ADA-positive (36.4%) and ADA-negative (34.8%) patients, as was the incidence of grade 3/4 AEs (63.6 and 56.1%, respectively). Overall, 7/11 (63.6%) ADA-positive and 29/66 (43.9%) ADA-negative patients experienced ≥ 1 immune-related AESI. Interpretation of any effect of ADA on AE/AESI incidence or severity in pediatrics was limited by the low number of ADA-positive patients.

The PK and safety profile was generally comparable between ADA-positive and ADA-negative patients. The relationship between pediatrics and young adults in terms of demographics, disease, immune status, and genetics that could influence ADA production remains unknown given the small size of the ADA-positive population.

## Discussion

This is the first report describing quantitative clinical pharmacology findings of a PD-1−/PD-L1-based ICI in pediatric patients. Atezolizumab exposures in pediatrics using weight-adjusted dosing were ~ 20% lower than in young adults receiving a flat dose; this is not considered clinically meaningful as both groups showed a substantial overlap and achieved the target trough concentration of 6 μg/mL [27, 28]. The popPK model adequately described the data after estimating parameters using pediatric data. Typical CL and V1 estimates were generally similar between pediatric (0.217 L/day, 3.01 L) and adult (0.200 L/day, 3.28 L) models, indicating appropriate scaling by body weight. Between-subject, proportional residual, and additive residual variability were consistent with the adult model. The magnitude of covariate effects was also similar to adults, except for sex, which was possibly confounded by weight. Children had approximately 20% lower AUC. These differences were not associated with a decrease in atezolizumab concentration below the therapeutic target level.

The body weights of young adults ≥ 18 years were relatively lower than those in the phase I atezolizumab adult studies; PK observations for these patients were consistent with what would be expected for adults with body weights ranging in the low end. The allometric coefficient on CL for weight was approximately 0.8, consistent with the typical accepted allometric value of 0.75 [25]. As CL was less than weight-proportional for a weight-based dosing regimen, exposure will be slightly less for patients of lower weight. An increase in CL that is less than proportional with weight has also been shown for other mAb, illustrating the importance of interpreting exposure metrics in pediatric and adult populations [34].

Trough exposures of atezolizumab in pediatric patients were generally consistent with those reported in adults and were above the target exposure of 6 μg/mL [29]. Median troughs in pediatric patients were ~ 10-fold and ~ 20-fold higher at cycle 1 and steady-state, respectively, compared with target exposure. While the tumor biology and microenvironment could be different in pediatric patients compared with adults, such exposure is expected to achieve comparable efficacy to that observed in adults. All pediatric patients achieved exposures within the prior realm of clinical experience compared with the phase I investigation of atezolizumab in adults (study PCD4989g; NCT01375842), which demonstrated clinical activity at doses ranging from 1 to 20 mg/kg [35].

Exposures of atezolizumab in the two infants in the study were lower than in young children; age-dependent physiologic processes may govern the disposition of atezolizumab in this specific population. Infants have a greater extracellular fluid content, higher total body V, greater cardiac output, and faster rate of perfusion into leaky tissues compared with older children and adults, in addition to FcRn binding differences [36]. Small changes in these properties have confirmed large differences in exposure of mAbs in physiologically-based PK models [37, 38]. Additional clinical investigation is warranted to determine an appropriate dose in infants.

Flat relationships of exposure-safety have been observed across multiple anti-PD-1/PD-L1 agents in adults, but no exposure-safety analyses for these agents have been reported in pediatric patients [39]. AE reporting of atezolizumab in pediatric patients was conducted in the same manner as in adults, except for infusion-related reaction AESI methodology, in which a wide basket search of a pre-specified infusion-related reaction/hypersensitivity Medical Dictionary for Regulatory Activities term reported within 24 h of infusion is used for pediatric patients versus a two-preferred-term search methodology of infusion-related reaction and cytokine release syndrome in adults. The distribution of AE and AESI was similar across age groups in our study, with no new safety signals identified. Safety findings of atezolizumab in pediatric patients were consistent with pooled phase I/II data in adults (*n* = 513) in the IMvigor210 (NCT02108652) and PCD4989g studies, which identified a flat exposure-safety profile [27]. Our observations in pediatric patients are also similar to other single-agent anti-PD-1/PD-L1 phase III studies in adults, which showed high-grade toxicities occurring in up to 34% of patients [40, 41]. Although numerically higher exposures were observed in young adult patients aged ≥ 18 years relative to patients aged < 18 years, the frequency and intensity of AEs were similar between the two groups. The range of atezolizumab exposure in pediatric patients falls below the highest exposure for which acceptable tolerability has been demonstrated in adults receiving 20 mg/kg in study PCD4989g. The overall safety profile of atezolizumab in the pediatric population was consistent with adults and confirmed a lack of relationship with exposure following the 15 mg/kg q3w regimen.

Our popPK investigation revealed that the ADA effect on atezolizumab clearance was similar between pediatric and young adult patients (23% increase in pediatric patients vs. 16% increase in young adults). The treatment-emergent ADA incidence is comparable to historical data in adults with ADA-positivity (ranging from 13 to 48% for atezolizumab [42]). Despite the limited number of ADA-positive pediatric and young adult patients, and given the relatively small sample size, mean differences in atezolizumab exposure for ADA-positive patients appeared to be similar to, and within the variability and range of, those observed in ADA-negative patients. No clinically meaningful impact on exposure or safety by ADA response to atezolizumab in pediatric patients was observed, although interpretation is limited by the low number of ADA-positive versus ADA-negative patients.

The limited number of responders (4/87) precluded an exposure-response assessment of outcomes. The majority of patients received atezolizumab for < 2 months, and discontinued early due to disease progression. Much has yet to be learned about the use of ICI in pediatric patients with cancer with respect to exposure-response outcomes [43]. In addition to the approval of nivolumab for pediatric patients with refractory HL, multiple other pediatric approvals exist including avelumab for merkel cell carcinoma, and pembrolizumab for recurrent locally advanced or metastatic merkel cell carcinoma, refractory classical HL, refractory primary mediastinal large B-cell lymphoma, and unresectable or metastatic microsatellite instability-high or mismatch repair-deficient tumors [44–47]. Ultimately, it may be challenging to provide complete remission of heavy-burden pediatric cancers with ICI monotherapy [48]. However, the understanding of PK/PD and dose selection in pediatric trials to optimize effective immunotherapies and their combinations to improve patient outcomes will be a key component in treating these complex cancers.

## Conclusion

This manuscript reports quantitative investigations of atezolizumab exposure, relationships of exposure with safety, and immunogenicity findings in pediatric patients. The pediatric and young adult popPK model adequately described the data and revealed similar PK parameters and covariate effects in pediatric patients compared with adults. Exposure distributions of atezolizumab largely overlapped between age groups with all pediatric patients achieving exposures in the range previously demonstrated in adult dose-ranging trials. The exposure profile and safety summary of atezolizumab suggest 15 mg/kg q3w as an appropriate posology sufficient to support possible future development in the pediatric population.

## Supplementary information

**Additional file 1: Figure S1.** Distribution of body weight in children, adolescents, and young adults receiving atezolizumab. Patients aged < 18 years (*n* = 69) received a 15 mg/kg q3w dose, while those aged ≥ 18 years (*n* = 18) received a 1200 mg q3w dose. Median weights: 38.9 kg for 15 mg/kg q3w and 61.0 kg for 1200 mg q3w. Abbreviation: *q3w* every 3 weeks.

**Additional file 2: Figure S2.** Scatterplot of random effects of (**A**) clearance and (**B**) volume of distribution parameters by body surface area in pediatric patients. Solid circles represent estimates in 69 patients receiving 15 mg/kg intravenous atezolizumab q3w. The blue line represents a loess trend. Abbreviations: *BSA* body surface area, *CL* clearance, *q3w* every 3 weeks, *V1* volume of the central compartment.

**Additional file 3: Figure S3.** Scatterplot of individual atezolizumab (**A**) clearance and (**B**) volume of distribution versus body weight in pediatric and young adult patients. Solid circles represent estimates in 87 patients, blue circles depict pediatric patients receiving 15 mg/kg q3w (*n* = 69) up to a maximum of 1200 mg, while red circles depict young adult patients receiving 1200 mg q3w (*n* = 18). The line represents a linear regression while the shaded area reflects the standard error of the regression line for the mean prediction. Abbreviation: *q3w* every 3 weeks.

**Additional file 4 Table S1.** Geometric mean (CV% geo mean) peak and trough concentration of atezolizumab (μg/mL) by ADA status across multiple cycles in patients aged 2 to < 12 years, 12 to < 18 years, or ≥ 18 years of age receiving 15 mg/kg or 1200 mg atezolizumab q3w.

## Data Availability

Qualified researchers may request access to individual patient level data through the clinical study data request platform (www.clinicalstudydatarequest.com). Further details on Roche’s criteria for eligible studies are available here (https://clinicalstudydatarequest.com/Study-Sponsors/Study-Sponsors-Roche.aspx). For further details on Roche’s Global Policy on the Sharing of Clinical Information and how to request access to related clinical study documents, see here (https://www.roche.com/research_and_development/who_we_are_how_we_work/clinical_trials/our_commitment_to_data_sharing.htm).

## Abbreviations

ADA: Anti-drug antibody
AE: Adverse event
AESI: Adverse events of special interest
AUC: Area under the curve
BSA: Body surface area
CI: Confidence interval
CL: Clearance
C_max_: Maximum concentration
C_min_: Minimum concentration
CWRES: Conditional weighted residuals
ELISA: Enzyme-linked immunosorbent assay
FcRn: Neonatal Fc receptor
HL: Hodgkin lymphoma
ICI: Immune checkpoint inhibitors
IQR: Interquartile range
LOQ: Lower limit of quantification
mAb: Monoclonal antibody
NHL: Non-Hodgkin lymphoma
ORR: Objective response rate
PD-1: Programmed cell death-1
PD-L1: Programmed cell death-ligand 1
PFS: Progression-free survival
PK: Pharmacokinetic
popPK: Population-PK
Q: Inter-compartmental clearance
q3w: Every 3 weeks
RECIST: Response Evaluation Criteria in Solid Tumors
V: Volume of distribution
V1: Volume of the central compartment
V2: Volume of the peripheral compartment
