# Supplementary material for: Population pharmacokinetics, exposure-safety, and immunogenicity of atezolizumab in pediatric and young adult patients with cancer
Source: J Immunother Cancer. 2019 Nov 21;7:314. doi: 10.1186/s40425-019-0791-x (PMC6868826; doi:10.1186/s40425-019-0791-x)
Supplement: Supplementary file 1 — Additional file 1: Figure S1. Distribution of body weight in children, adolescents, and young adults receiving atezolizumab. Patients aged < 18 years (n = 69) received a 15 mg/kg q3w dose, while those aged ≥ 18 years (n = 18) received a 1200 mg q3w dose. Median weights: 38.9 kg for 15 mg/kg q3w and 61.0 kg for 1200 mg q3w. Abbreviation: q3w every 3 weeks. [file 40425_2019_791_MOESM1_ESM.docx]

**Additional file 1: Figure S1** Distribution of body weight in children, adolescents, and young adults receiving atezolizumab.

Patients aged < 18 years (*n* = 69) received a 15 mg/kg q3w dose, while those aged ≥ 18 years (*n* = 18) received a 1200 mg q3w dose. Median weights: 38.9 kg for 15 mg/kg q3w and 61.0 kg for 1200 mg q3w. Abbreviation: *q3w* every 3 weeks
