# Supplementary material for: Population pharmacokinetics, exposure-safety, and immunogenicity of atezolizumab in pediatric and young adult patients with cancer
Source: J Immunother Cancer. 2019 Nov 21;7:314. doi: 10.1186/s40425-019-0791-x (PMC6868826; doi:10.1186/s40425-019-0791-x)
Supplement: Supplementary file 2 — Additional file 2: Figure S2. Scatterplot of random effects of (A) clearance and (B) volume of distribution parameters by body surface area in pediatric patients. Solid circles represent estimates in 69 patients receiving 15 mg/kg intravenous atezolizumab q3w. The blue line represents a loess trend. Abbreviations: BSA body surface area, CL clearance, q3w every 3 weeks, V1 volume of the central compartment. [file 40425_2019_791_MOESM2_ESM.docx]

**Additional file 2: Figure S2** Scatterplot of random effects of (**A**) clearance and (**B**) volume of distribution parameters by body surface area in pediatric patients.

Solid circles represent estimates in 69 patients receiving 15 mg/kg intravenous atezolizumab q3w. The blue line represents a loess trend. Abbreviations: BSA body surface area, CL clearance, q3w every 3 weeks, V1 volume of the central compartment
