# Supplementary material for: Population pharmacokinetics, exposure-safety, and immunogenicity of atezolizumab in pediatric and young adult patients with cancer
Source: J Immunother Cancer. 2019 Nov 21;7:314. doi: 10.1186/s40425-019-0791-x (PMC6868826; doi:10.1186/s40425-019-0791-x)
Supplement: Supplementary file 3 — Additional file 3: Figure S3. Scatterplot of individual atezolizumab (A) clearance and (B) volume of distribution versus body weight in pediatric and young adult patients. Solid circles represent estimates in 87 patients, blue circles depict pediatric patients receiving 15 mg/kg q3w (n = 69) up to a maximum of 1200 mg, while red circles depict young adult patients receiving 1200 mg q3w (n = 18). The line represents a linear regression while the shaded area reflects the standard error of the regression line for the mean prediction. Abbreviation: q3w every 3 weeks. [file 40425_2019_791_MOESM3_ESM.docx]

**Additional file 3: Figure S3** Scatterplot of individual atezolizumab (**A**) clearance and (**B**) volume of distribution versus body weight in pediatric and young adult patients.

Solid circles represent estimates in 87 patients, blue circles depict pediatric patients receiving 15 mg/kg q3w (n = 69) up to a maximum of 1200 mg, while red circles depict young adult patients receiving 1200 mg q3w (n = 18). The line represents a linear regression while the shaded area reflects the standard error of the regression line for the mean prediction. Abbreviation: q3w every 3 weeks
