# Supplementary material for: Population pharmacokinetics, exposure-safety, and immunogenicity of atezolizumab in pediatric and young adult patients with cancer
Source: J Immunother Cancer. 2019 Nov 21;7:314. doi: 10.1186/s40425-019-0791-x (PMC6868826; doi:10.1186/s40425-019-0791-x)
Supplement: Supplementary file 4 — Additional file 4 Table S1. Geometric mean (CV% geo mean) peak and trough concentration of atezolizumab (μg/mL) by ADA status across multiple cycles in patients aged 2 to < 12 years, 12 to < 18 years, or ≥ 18 years of age receiving 15 mg/kg or 1200 mg atezolizumab q3w. [file 40425_2019_791_MOESM4_ESM.docx]

**Additional file 4: Table S1** Geometric mean (CV% geo mean) peak and trough concentration of atezolizumab (ug/mL) by ADA status across multiple cycles in patients aged 2 to < 12 years, 12 to < 18 years, or ≥ 18 years of age receiving 15 mg/kg or 1200 mg atezolizumab q3w

| Visit | ADA-negative  patients, *n* | Geometric mean  (CV% geo mean) | ADA-positive  patients, *n* | Geometric mean  (CV% geo mean) |
| --- | --- | --- | --- | --- |
| Cycle 1 day 1 post-dose | 60 | 350 (29.2) | 11 | 384 (25.5) |
| Cycle 2 day 1 pre-dose | 61 | 62.5 (46.7) | 10 | 57.0 (54.0) |
| Cycle 3 day 1 pre-dose | 35 | 99.6 (47.8) | 4 | 64.4 (59.5) |
| Cycle 4 day 1 pre-dose | 30 | 112 (44.8) | 3 | 87.1 (70.0) |
| Cycle 4 day 1 post-dose | 30 | 412 (60.9) | 3 | 418 (15.5) |

Abbreviations: *ADA* anti-drug antibodies, *q3w* every 3 weeks

Given the small sample size, exposure relationships by treatment-emergent ADA status is presented across all ages and includes a combination of patients aged 2 to < 12 years, 12 to < 18 years, and ≥ 18 years. Patients aged < 18 years received a 15 mg/kg dose of atezolizumab q3w while patients aged ≥ 18 years received a flat 1200 mg dose of atezolizumab q3w
